# Exploring the impact of autumn color and bare tree landscapes in virtual environments on human well-being and therapeutic effects across different sensory modalities

**DOI:** 10.1371/journal.pone.0301422

**Published:** 2024-04-18

**Authors:** Menglei Yin, Kankan Li, Zhiman Xu, Rui Jiao, Wenzhi Yang

**Affiliations:** College of Landscape Architecture and Art, Northwest Agriculture and Forestry University, Xianyang, China; University of Bucharest, Faculty of Biology, ROMANIA

## Abstract

In recent years, there has been a growing awareness of the potential health benefits of the natural environment for human well-being. Given the fast-paced nature of contemporary lifestyles, research into the use of virtual environments as a means to provide various seasonal landscapes has gained increasing importance. **Objective:** The aim of this study is to investigate the impact of different sensory modes on the preferences and therapeutic effects of virtual autumn landscapes on university campuses. **Methods:** In this study, 320 participants, with an average age of 21.11 years (±1.21 years), were exposed to virtual environments featuring autumn color landscapes and bare tree landscapes using visual, auditory, and combined conditions. A control group was included for comparison. Differences in participants’ physiological indicators (EEG, heart rate) and psychological measures (POMS, PANAS, SVS, ROS) were analyzed, with the use of the Holm correction (*P* < 0.05). **Results:** (1) Autumn virtual landscapes with color had a superior therapeutic effect. (2) There were significant differences in the therapeutic effects of different sensory modes within the same season’s landscape categories, suggesting that incorporating additional sensory dimensions may enhance therapeutic outcomes. **Conclusion:** Based on the study’s findings, we recommend that when designing therapeutic environments, attention should be given to seasonal variations and the integration of various sensory modes to optimize therapeutic results.

## 1 Introduction

### 1.1 The impact of campus landscapes on individual physical and mental recovery

Natural environments, as integral components of ecosystems, fulfill diverse ecological functions, including air quality improvement and water resource conservation [1]. Engaging in activities within natural settings is advantageous for both physical and mental well-being [1]. Campus landscapes serve as crucial avenues for university students to connect with nature frequently [2], contributing to heightened emotional experiences, emotional quality, stress reduction, and enhanced self-satisfaction [3].

Although there has been extensive research on the relationship between [4] campus landscapes and the restoration of physical and mental well-being, further investigation is warranted to delve into the impact of seasonal variations in the campus’s natural environment on healing outcomes.

### 1.2 The impact of autumn landscape colors and bare trees on individual physical and mental recovery

In studies focusing on the benefits of natural environments, there is typically an emphasis on the advantages of green vegetation [4–6]. However, it is worth noting that seasonal variations can also significantly influence individual landscape preferences [7]. For instance, during the autumn season, individuals tend to exhibit a stronger preference for vividly colored landscape elements, such as maple leaves, whereas preferences for bare trees are less pronounced in other seasons [8–10]. Even during winter, when snowy landscapes become more popular, prior research suggests that bare trees still contribute to the restoration process [11, 12]. Importantly, earlier studies have indicated that bare tree landscapes similarly possess restorative effects on individuals, although the role of seasonal variations has not received sufficient attention [13]. Therefore, our study focuses on the seasonal changes in plants, with a particular emphasis on the restorative effects of barren tree landscapes following the conclusion of autumn. We aim to elucidate the differences in restorative effects between autumn-colored plants and bare trees, with the ultimate goal of increasing appreciation and attention to post-autumn landscapes, including the beauty of leafless trees [14, 15].

### 1.3 The influence of various sensory dimensions on human physical and mental recovery

In terms of research methodologies, most studies related to landscape restoration effects have traditionally focused on the visual restorative effects of landscape vegetation [5]. They emphasize how the aesthetic features of natural landscapes influence emotions and well-being. However, as research advances, scholars have come to recognize the multidimensional nature of the perceptual system, where various senses intertwine and collectively influence the physical and mental states of individuals. Consequently, some researchers have shifted their focus towards multidimensional sensory dimensions, including visual, auditory, and olfactory experiences, to investigate the therapeutic effects of landscape vegetation [16–18].

Therefore, we consider the transition from a single sensory dimension to multidimensional sensory modes to explore the variations in the physical and mental recovery effects on participants influenced by different sensory modes.

### 1.4 The application of VR technology in landscape restoration

Field surveys are susceptible to uncontrollable factors and unexpected events, making tools such as photos, Virtual Reality (VR), and eye-tracking devices increasingly essential in landscape perception and preference research [6, 11, 13].

With the rapid advancement of VR technology, an increasing number of scholars are turning their attention to VR-based natural landscape therapy [17]. This technology simulates multisensory experiences in natural environments, enabling the study of people’s preferences for natural settings and their potential in stress reduction. By creating immersive experiences within virtual environments, VR technology achieves enhanced therapeutic outcomes [19–21].

Moreover, virtual reality can replicate seasonal landscape changes [22]. Research has shown that viewing natural imagery contributes to physiological stress reduction and emotional relaxation, making virtual reality an alternative approach to experiencing nature for restoration purposes [11, 23–25].

Therefore, our research places a stronger emphasis on the effects of landscapes on individuals in virtual environments while delving into the distinctions in the restorative outcomes of virtual landscape experiences.

### 1.5 Research objectives and hypotheses

Our study is rooted in Attention Restoration Theory (ART) [26], Stress Reduction Theory (SRT) [27], and other theories relevant to restorative landscapes. It draws upon the works of scholars such as Rachel Kaplan, Stephen Kaplan, and Roger Ulrich.

Rachel Kaplan posits that natural landscapes play a crucial role in restoring human attention and cognition by providing simple, soothing sensory stimuli that reduce cognitive load, alleviate physical and mental stress, enhance responsiveness, and renew cognitive resources. Roger Ulrich points out that natural environments can assist individuals in stress reduction and psychological equilibrium.

In summary, our research explores the therapeutic effects of autumn-colored foliage and bare tree landscapes in virtual environments, considering multi-sensory dimensions for differentiation. Our goal is to unveil the benefits of both colorful and colorless bare tree landscapes for mental and physical recovery [28].

This is of significant importance in elevating people’s appreciation for different seasonal landscapes.

This study posits the following hypotheses:

Hypothesis 1: In virtual environments, even during autumn, landscapes featuring bare trees alone exhibit a restorative effect and offer certain benefits for physical and psychological well-being.
Hypothesis 2: Visual and auditory stimuli within landscapes significantly impact physiological and psychological responses. It is noteworthy that when these stimuli are combined, restorative effects are more pronounced, proving more effective than individual sensory stimuli.

## 2 Research methodology

### 2.1 Materials and methods

#### 2.1.1 Participants

**Fig 1:**
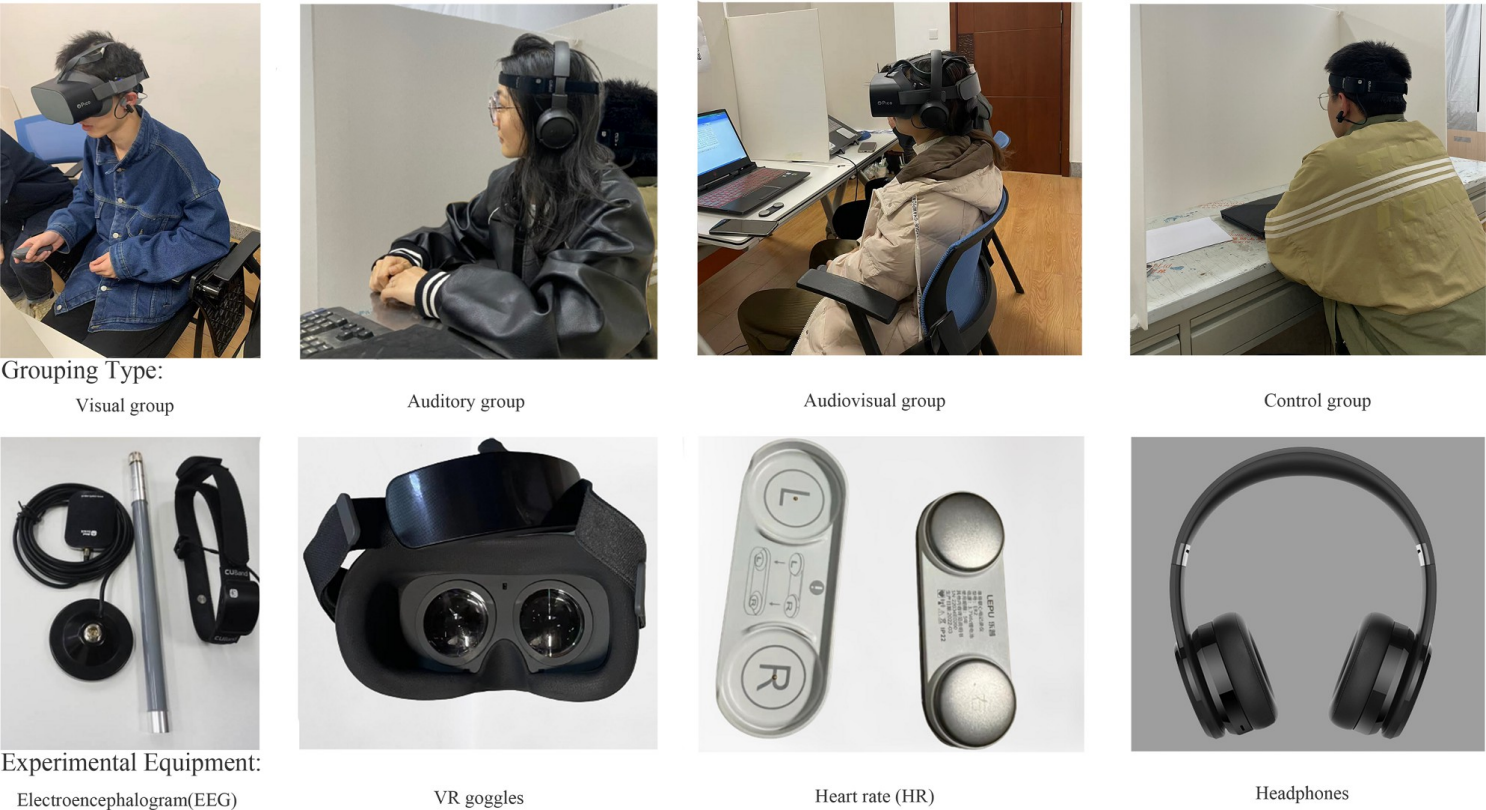
Group types and experimental equipment.

In December 2022, we randomly recruited 320 volunteers from various disciplines at Northwest A&F University, who had no history of mental disorders and had normal vision and hearing, to participate in the experiment. Their average age was 21.11 years (±1.21 years). The participants included 140 males (average age 21.16 ± 1.80 years; 43%) and 180 females (average age 21.96 ± 1.50 years; 56%). Before the experiment, each volunteer was assigned a unique identifier for later identification and was thoroughly informed to avoid vigorous physical activities, smoking, and alcohol consumption the day before the experiment. Our volunteer recruitment process and requirements strictly followed the ethical standards for scientific experiments set by the Psychological Center at Northwest A&F University and underwent evaluation by relevant experts. To prevent the Hawthorne effect [29], we did not disclose the research objectives to the volunteers in advance. Instead, we randomly assigned volunteers to different experimental groups. These experimental groups included a control group, a visual group (where participants were provided with clear explanations of the experimental procedure before it began, and an experimenter silently guided the operation, recording relevant experimental data. During the experiment, participants in the visual group wore head-mounted virtual reality (VR) goggles to eliminate potential interference factors), an auditory group (where auditory stimuli were delivered through professional headphone equipment, and participants faced three whiteboards, thus eliminating other influencing factors), and an audiovisual group (Fig 1).

#### 2.1.2 Study sites

The experiment was conducted in the Landscape Architecture Laboratory at Northwest A&F University. The experimental site covered an area of 12 square meters with dimensions of 4 meters (length) × 3 meters (width) × 3.1 meters (height). To eliminate any unnecessary visual disturbances, we used PVC whiteboards to isolate the test area from the surrounding environment. The experiment took place from 9 a.m. to 11 a.m. The indoor temperature was maintained at a comfortable level of 21.5°C, and the relative humidity ranged from 40% to 60%, ensuring no external noise interfered with the experiment.

#### 2.1.3 Experimental materials

**Fig 2:**
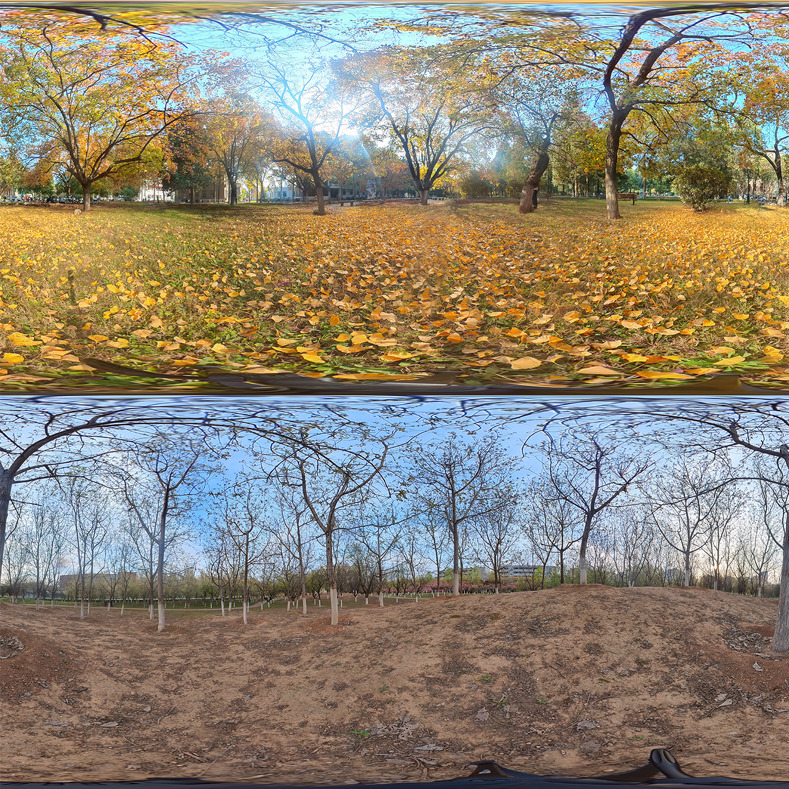
Experimental materials.

The audiovisual experiment materials were recorded between 9:00 AM and 11:00 AM in the morning of October 2022. The visual stimulus materials primarily consisted of 3-minute VR panoramic videos, captured using the Insta360 ONE X2 panoramic camera. These videos showcased the vibrant autumn scenes on the campus, including the autumn bare tree landscape. The images and panoramic photos were taken under mild, windless conditions, from a viewer’s perspective at a height of 1.4 meters [30, 31]. Twenty panoramic photos were evaluated by five landscape architecture experts. Based on factors such as color, space, brightness, contrast, and proportion, they selected the top-ranking color and bare tree panoramic photos (Fig 2). The resolution of the panoramic photos was 6080 × 3040 pixels, allowing participants to experience an immersive real-world environment through VR goggles (Pico G2).

In terms of auditory stimuli, high-resolution recordings of bird songs and natural wind sounds were collected using a recorder with a 96 kHz sampling rate and 24-bit quantization. The recording location for these sounds matched the settings of the autumn color landscapes and single landscape in terms of geography and time. The sound recordings were played back at an approximately 50-decibel volume level through headphones (JBL TUNE 500BT). Five landscape architecture experts were invited to evaluate the auditory stimulus materials, considering criteria such as sound comfort, naturalness, realism, clarity, and other aspects. The average scores from these evaluations were used as the final assessment results.

### 2.2 Survey measures

Based on the experimental design, this study identified physiological and psychological indicators to quantify the impact of different autumn landscape photos and sounds on participants’ recovery. To meet the experimental requirements and utilize existing equipment, we selected electroencephalogram (EEG) α-wave and heart rate (HR) (recorded with Lepu electrocardiograph ER2, screenless version, and dynamic monitoring) as the physiological indicators for the experimental research. For psychological indicators, we employed four psychological measurement tools, namely the Profile of Mood States (POMS), the Positive and Negative Affect Schedule (PANAS), the Recovery Outcome Survey (ROS), and the Subjective Vitality Scale (SVS).

#### 2.2.1 Physiological measurements

When the human brain is engaged in specific activities, it generates biological signals known as brainwaves. Brainwaves serve as an objective indicator of physiological responses, reflecting the brain’s reactions to external stimuli. In this study, we continued to utilize a portable EEG device manufactured by NeuroSky, a U.S. company. The reference electrode was placed on the left forehead of the participants, while the reference electrode employed an earlobe approach on the right. By applying NeuroSky’s ThinkGear™ technology, we amplified the raw EEG signals, filtered environmental noise, and mitigated interference from muscle tissue movement. We further processed the brainwave data using the eSense™ algorithm, enabling us to obtain various types of alpha waves (including low-frequency alpha1 and high-frequency alpha2) for each participant per second throughout the experiment.

Previous research has indicated a close relationship between alpha waves and an individual’s psychophysiological stress and emotional states. Higher alpha wave values are associated with greater physiological relaxation and increased overall relaxation levels. Hence, in this study, we employed alpha waves as the physiological indicator for college students [24, 32–34].

In scientific research, heart rate is a commonly used physiological indicator [35]. In this study, we will continue to employ a heart rate monitoring device to record participants’ heart rate changes per second. Heart rate is one of the most fundamental physiological responses in the human body and is typically positively associated with emotions, stress, physical activity, and more. In this research, heart rate will be recorded and utilized as a significant physiological indicator for the analysis of participants’ psychological responses and emotions. It will serve as a valuable basis and reference for our research findings. Therefore, heart rate is considered one of the physiological indicators for university students in this study.

#### 2.2.2 Psychological measures

POMS (Profile of Mood States): The Profile of Mood States is a questionnaire used to measure six distinct emotional states, including tension-anxiety, depression-dejection, anger-hostility, fatigue, confusion, and vigor. The questionnaire is effective, (The questionnaire has been tested for suitability among Chinese volunteers Approval Number: NWAFU20220910) and this study employed a 40-item version (using a 5-point Likert scale ranging from 0—not at all to 5—extremely) [36].

PANAS (Positive and Negative Affect Schedule): The Positive and Negative Affect Schedule (PANAS) is a questionnaire designed to measure two types of emotional effects: positive and negative. It is a reliable questionnaire consisting of a total of 20 items, and in this study, we used a 5-point Likert scale [37].

ROS (Recovery Outcome Survey): The Recovery Outcome Survey evaluates the recovery effects of each environment, including six components. The scale has demonstrated validity and reliability [38].

SVS (Subjective Vitality Scale): The Subjective Vitality Scale comprises six items that assess vitality. This scale is known for its reliability [39].

All questionnaires were provided in Chinese. If participants encountered any unfamiliar terms or questions, the experimenters were available to provide explanations. To assess the coherence and internal consistency of the data, we utilized Cronbach’s alpha to evaluate the questionnaires.

### 2.3 Experimental procedure

The experiment was conducted in the laboratory of the College of Landscape Architecture at Northwest A&F University in December 2022. To minimize participants’ unfamiliarity and excitement in the presence of testing equipment, we required them to arrive five minutes early at the experimental site and maintain a calm state of mind in their seats [22]. Experimenters explained the experimental procedure to the volunteers without revealing the research’s purpose. Furthermore, before the formal experiment, participants were asked to wear the EEG equipment in advance to reduce discomfort during the equipment-wearing process and improve the stability of EEG data [7].

**Fig 3:**
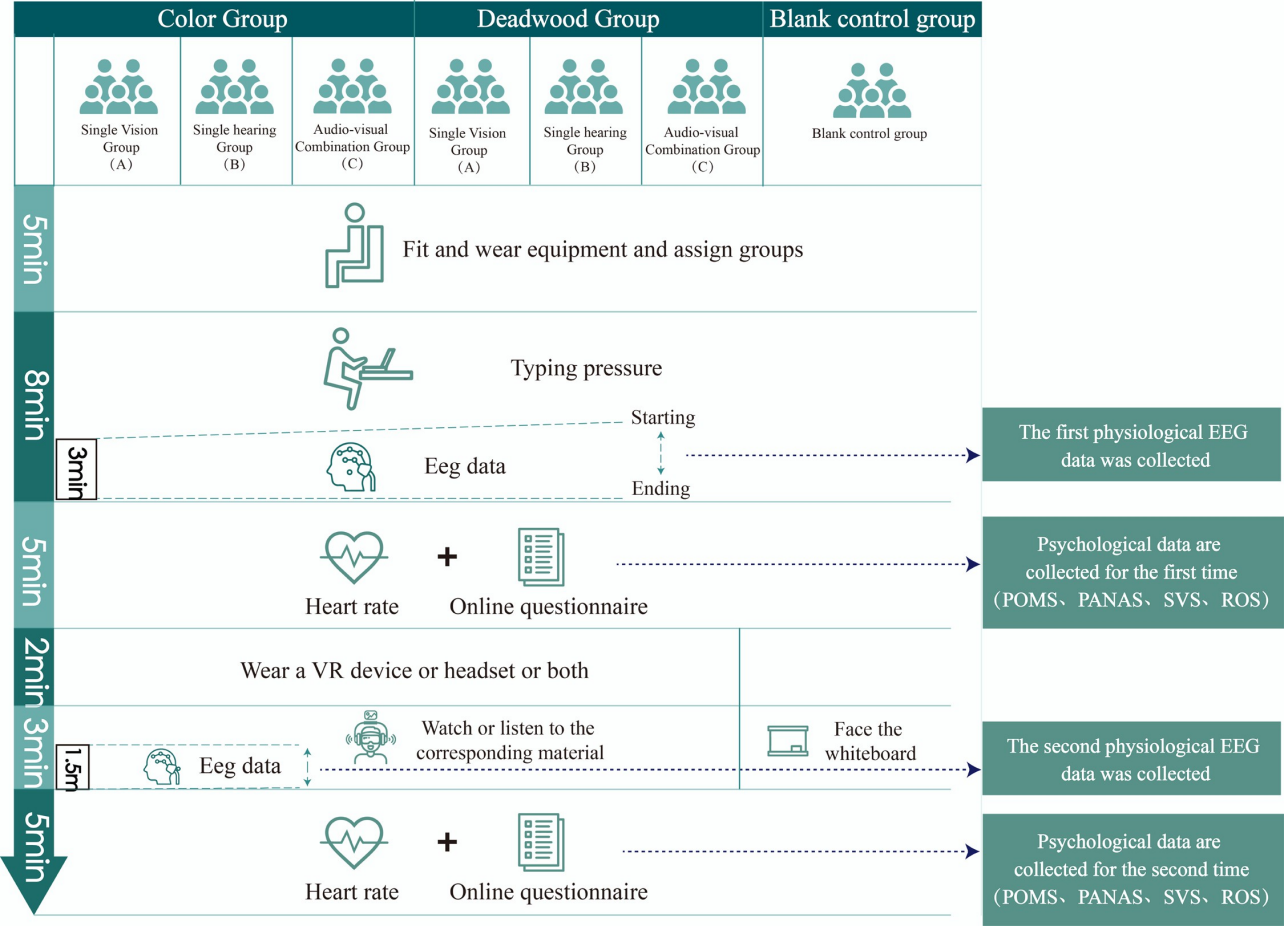
Experimental procedure.

We employed a random group allocation method to assign participants to three groups: the Color Group (scenes with rich colors of autumn landscape plants), the Bare Tree Group (scenes depicting leafless post-autumn landscape plants), and the Blank Classroom (Fig 3). The experiment comprised two phases. In the first phase, all group participants underwent stress induction. Participants from all three groups were required to complete a typing test within 8 minutes, with data recorded for the final three minutes. (To apply stress pressure was to make the effects of our experiment more pronounced. This phase was reviewed and approved by the Northwestern Agricultural and Forestry University’s Psychological Committee Center, meeting the experimental requirements, with approval number NWAFU: 202210078) After the test, we collected pre-test EEG data, heart rate data, and guided participants to complete prepared questionnaires online by scanning a QR code (After being examined and approved by the Committee of the Center for Psychological Development and Education at Northwest A&F University, with approval number NWAFU20220916).

In the second phase, experimenters began to fit VR devices for participants in all three groups. During this process, participants received materials from the devices, involving either single visual, single auditory, or audio-visual combination stimuli, with a duration of 3 minutes. We recorded data from the final 90 seconds as the valid data for this experiment. Selection of data from the last 90 seconds was based on prior research, ensuring that participants had reached a stable state and received a sufficient duration of stimuli. Moreover, data during this time frame are typically more stable because all participants received stimuli within the same time frame, reducing interference from variables. After the experiment concluded, we spent 5 minutes collecting post-test EEG data, heart rate data, and guided participants to complete prepared questionnaires online by scanning a QR code (Fig 3). Ethical Statement: All participants provided informed consent before participating in the study. Publication of this paper has obtained written informed consent from the patients. This research was conducted in accordance with the Helsinki Declaration, and the experimental procedures, which were reviewed and approved by the Psychological Development and Education Center of Northwest A&F University, adhered to ethical standards. The approval code is 33/2022, and it was granted on September 5, 2022.

### 2.4 Data analysis

In this study, all data were subjected to statistical processing using Microsoft Excel 2016, and statistical analyses were performed using SPSS 22.0 [40]. Physiological and psychological responses of participants under different stimulus conditions (Color Group, Bare Tree Group, from single dimension to multi-dimensions) and the control group were analyzed through one-way analysis of variance (ANOVA) and paired t-tests, with Holm correction applied (*P* < 0.05). Cohen’s *d* was utilized to represent the effect size of t-tests (effect magnitude) [41]. Finally, data visualization was conducted using Photoshop 2019 and Prismchs.

## 3 Research findings

### 3.1 Impact of autumn color landscapes on human recovery

#### 3.1.1 Baseline homogeneity test for initial value table

To begin, the pre-experiment data of physiological indicators, including electroencephalograms (EEG) and heart rate, as well as psychological indicators (POMS, PANAS, SVS, ROS), were collected from the six groups of participants. Single-factor analysis of variance was conducted to assess their homogeneity. The results indicated no significant differences in EEG wave values, heart rate, and psychological indicators (POMS, PANAS, SVS, ROS) among the participants across the six experimental sessions. The exception was the EEG wave values in the Bare Tree Group—Audio-Visual condition, suggesting that these data could be considered as the initial baseline for the physiological indicators, encompassing EEG and heart rate values (S1 and S2 Tables).

#### 3.1.2 Physiological effects of autumn color plants on human recovery

In this study, paired t-tests were employed to examine the changes in participants’ brainwave patterns and heart rates before and after experiencing autumnal colored plants in a virtual reality environment. The results indicate that, following the experience of autumnal colored plants through virtual reality devices, significant alterations were observed in the alpha-1 (α1) and alpha-2 (α2) brainwave patterns for both the control group and the experimental group, across three different modes of stimulation (visual only, auditory only, and audio-visual combined). This suggests that autumnal colors have a certain stress recovery effect on individuals.

**Fig 4:**
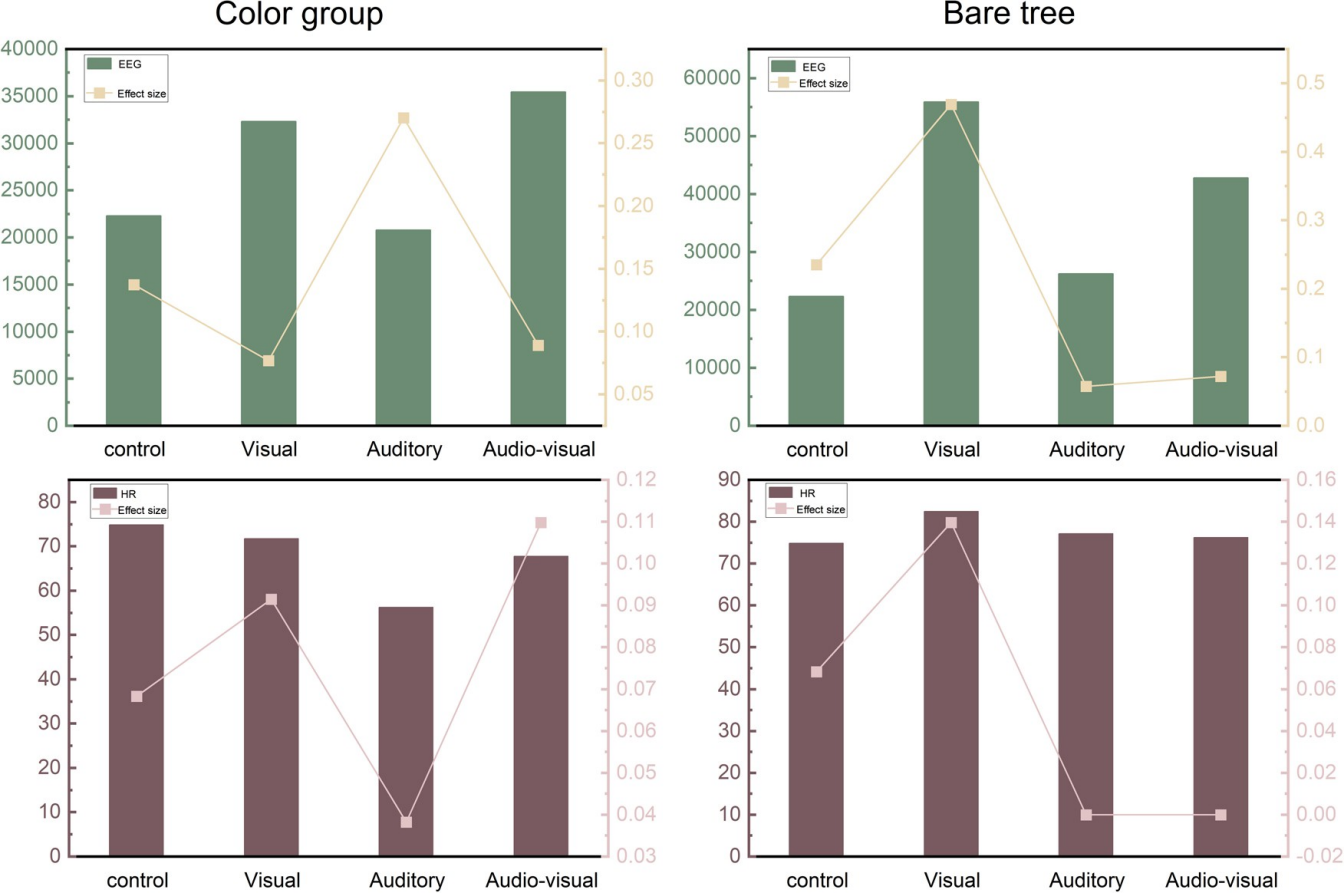
The color group and the bare tree group’s physiological responses.

Analyzing the effect size indicators, it is evident that the auditory stimulation group demonstrated a greater frequency of change in EEG values compared to the control group, while the visual and audio-visual conditions showed minimal differences in effect size. When comparing heart rate values to those of the control group, the auditory group exhibited a higher level of significance, while the audio-visual condition displayed a substantially larger difference in effect size. These findings not only provide a basis for a more profound understanding of the impact of visual and auditory stimuli on heart rate but also offer reference and inspiration for future related research and applications (Fig 4 and S3 Table).

#### 3.1.3 Psychological effects of autumn color plants on human recovery (Four questionnaires)

In this study, we utilized paired t-tests to examine changes in participants’ emotional ratings (assessed through POMS, PANAS, ROS, SVS) before and after experiencing autumnal colored plants in a virtual reality environment. The results indicate that, in terms of POMS scores, all groups exhibited significant differences compared to the control group, with the auditory group (*P* < 0.029) being the most significant, followed by the visual group (*P* < 0.033), and the audio-visual combined group (*P* < 0.033). Effect size indicators reveal substantial differences in all groups when compared to the control group, with the audio-visual condition displaying the greatest variance.

Concerning PANAS scores, all groups showed significant differences compared to the control group, with the audio-visual group (*P* < 0.016) being the most significant, followed by the visual group (*P* < 0.017) and the auditory group (*P* < 0.029). Effect size indicators again highlight substantial differences in all groups when compared to the control group, with the audio-visual condition displaying the greatest disparity.

The same approach was applied to the evaluation of ROS data, and significant differences were observed in all groups for various modes of stimulation, with the auditory group (*P* < 0.008) displaying the highest significance. Effect size indicators reveal considerable distinctions, particularly in the visual and auditory groups, when compared to the control group. This suggests that autumnal colored plants have a notable positive impact on individuals’ recovery.

**Fig 5:**
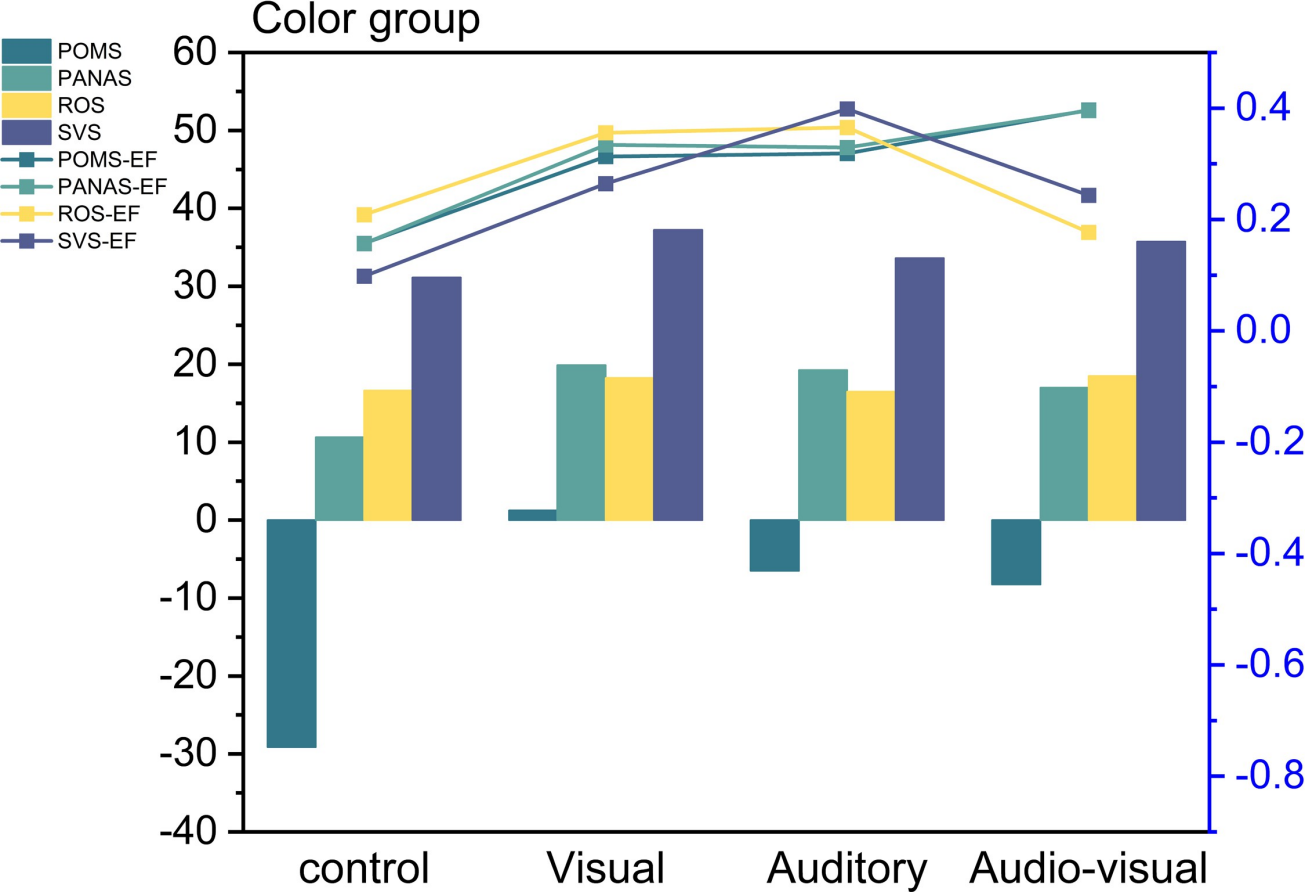
Physiological indicator changes in the color group.

Furthermore, the assessment of subjective vitality (SVS) scores revealed significant differences in all groups under various modes of stimulation, with auditory data exhibiting the most pronounced changes. Effect size indicators consistently indicate substantial disparities in all groups compared to the control group (Fig 5 and S4 Table).

### 3.2 Impact of autumn bare tree landscapes on human recovery

#### 3.2.1 Physiological effects of autumn bare tree landscapes on human recovery (EEG and heart rate)

In this study, we employed EEG and heart rate monitoring to examine changes in participants’ EEG and heart rate data before and after experiencing an autumnal bare tree landscape in a virtual environment. We assessed the fatigue recovery effects across different dimensions.

The results indicate that within the autumnal bare tree landscape, the single visual dimension exhibited the most significant recovery effects in terms of EEG data (α1 wave, *P* < 0.043; α2 wave, *P* < 0.067). Following this, the single auditory group displayed recovery effects, though a1 wave (*P* < 0.788) did not exhibit significance. In the case of audio-visual stimulation, the EEG data did not demonstrate statistical significance in terms of P-values, but effect size indicators suggest substantial differences between the visual and auditory groups when compared to the control group, with no differences observed in the audio-visual group.

Regarding heart rate, only minor significance was observed under visual stimulation, with no significant effects noted in the auditory and audio-visual conditions. Effect size indicators suggest minimal differences, primarily in the visual group. These findings provide valuable insights into the impact of different dimensions on human recovery (Fig 4 and S5 Table).

#### 3.2.2 Psychological effects of autumn bare tree landscapes on human recovery (Four emotional questionnaires)

In this study, we conducted an analysis of questionnaire data using variance and post hoc t-tests to examine the variations in the restorative effects of autumnal bare tree landscapes in a virtual environment across different dimensions. In the POMS questionnaire, all groups exhibited significant differences compared to the control group, with the auditory-only dimension displaying the most pronounced changes (*P* < 0.006), followed by the visual-only dimension (*P* < 0.016). However, the audio-visual dimension showed non-significant effects (*P* < 0.475). Effect size indicators indicate a considerable deviation for the audio-visual data from our initial predictions. Substantial differences are observed between the visual and auditory groups.

Regarding the PANAS emotional scale, the analysis of pre- and post-data revealed significant differences in the visual, auditory, and control groups. In contrast, the audio-visual dimension showed poorer effects, with values greater than those of the control group. Effect size indicators suggest a considerable deviation for the audio-visual data from our initial predictions, with substantial differences between the visual and auditory groups.

In the ROS recovery outcome scale, statistical data indicate that the values for the visual, auditory, and audio-visual groups are all significantly different from the control group. The visual-only group exhibited the most pronounced significance (*P* < 0.032), followed by the auditory group (*P* < 0.062). However, the statistical data for the other audio-visual combinations all had P-values greater than 0.05, rendering their results inconclusive. Effect size indicators suggest a considerable deviation for the audio-visual data from our initial predictions, with substantial differences between the visual and auditory groups.

**Fig 6:**
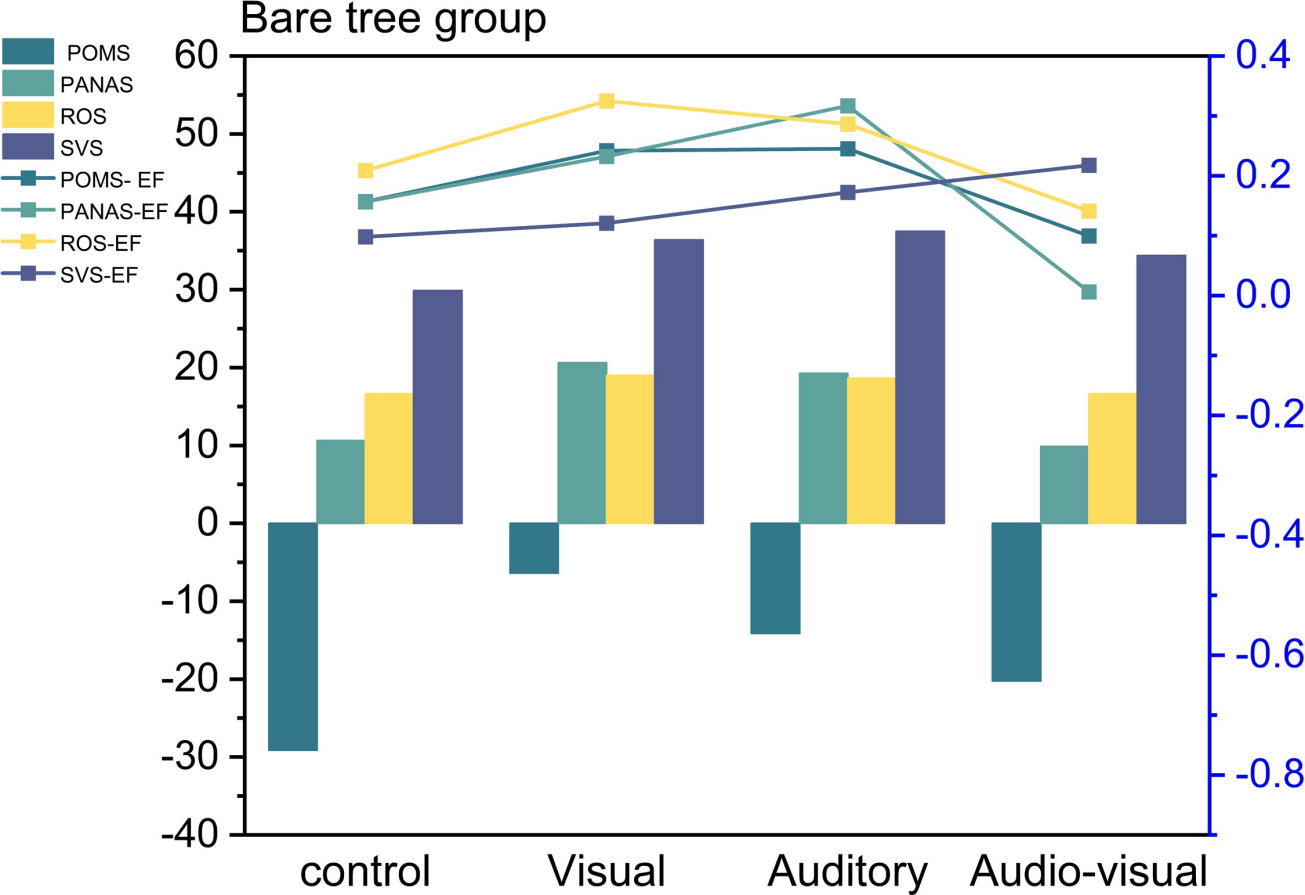
The psychological changes in the bare tree group.

In the SVS scale, while post-data showed significant differences compared to the control group, the audio-visual dimension exhibited the most pronounced effects (*P* < 0.035), followed by the auditory-only and visual-only dimensions, both with P-values greater than 0.5. Effect size indicators indicate substantial differences in all these data results when compared to the blank control group, with the audio-visual recovery distinction being the most prominent, aligning with our predictions (Fig 6 and S6 Table).

### 3.3 Impact on human recovery from a single to multidimensional perspective

#### 3.3.1 Physiological impact of perceived dimensions of autumn landscapes in a virtual environment on human recovery

In this study, we conducted a data analysis of EEG and heart rate before and after experiencing autumn landscapes in a virtual environment, ranging from single perspective to multidimensional perspective, using analysis of variance (ANOVA) and post hoc t-tests. The results indicate that in the α-wave of EEG, the visual group, the auditory color group, and the bare tree group exhibited significant differences, while the audio-visual combination group did not show statistical significance. Effect size analysis revealed no distinctions between the visual subgroup of the color group and the audio-visual subgroup of the color and bare tree groups. All other subgroups displayed substantial differences.

Regarding heart rate, all subgroups exhibited statistical significance, except for the audio-visual combination group. Effect size analysis indicated significant distinctions within the color subgroup of the audio-visual combination, with no clear distinctions among its subgroups (S7 Table).

#### 3.3.2 Psychological impact of perceived dimensions of autumn landscapes in a virtual environment on human recovery

Using the same testing methodology, we found that, in the POMS scale, the visual, auditory, and audio-visual combination groups exhibited significant differences when compared to the control group in both the color and bare tree subgroups. Notably, the auditory group displayed the highest significance. Effect size analysis reveals substantial differences in all groups except for the audio-visual combination group’s bare tree subgroup, which showed a comparatively smaller difference.

In the PANAS emotional recovery scale, all groups, except the bare tree subgroup within the audio-visual combination group, displayed significant differences when compared to the control group. Effect size analysis indicates substantial differences in all groups, with the auditory group showing the most significant difference.

For the ROS recovery scale, all groups, except for the color and bare tree subgroups within the audio-visual combination group, exhibited significant differences compared to the control group. Effect size analysis highlights significant distinctions in all groups, except for the audio-visual combination group’s color and bare tree subgroups, which showed no substantial differences.

In the SVS subjective vitality scale, all dimensional subgroups showed significance when compared to the control group. Effect size analysis indicates substantial distinctions in all dimensional subgroups, with significant differences particularly prominent in the audio-visual combination and auditory dimensions (S8 Table).

## 4 Discussion

### 4.1 Impact of seasonal landscape color types on recovery effects

#### 4.1.1 Enhanced recovery with diverse color types

Our study reveals that different types of colors within the same season have varying effects on recovery outcomes [42]. In general, within the autumn landscape, a rich array of colors contributes more to recovery compared to monochromatic scenes (such as the barren tree landscape). In the autumn environment, colorful landscapes positively impact psychological recovery, aiding in stress reduction and enhancing attention. Hence, there is a need to emphasize increasing the richness of colors within autumn landscapes, as it can assist people in stress alleviation, emotional regulation, and simultaneously improve work efficiency [43].

Green environments and diverse landscapes have a certain positive influence on work efficiency and cognitive tasks. Therefore, when designing indoor and outdoor workplaces, there should be an increased incorporation of green environments and colorful landscapes (e.g., gardens and courtyards) to enhance employees’ work efficiency and job satisfaction [6].

Research findings indicate that colorful and diverse landscapes in nature help individuals concentrate their attention and enhance their mental well-being [1]. Richer landscapes are often more appealing, effectively capturing visual attention, subsequently leading to recovery effects. Within the color evaluation of autumn landscapes, factors like the primary autumn color, color saturation, and brightness play essential roles. The primary autumn color significantly impacts visual preferences, while color saturation and brightness factors also contribute significantly to visual evaluations [44]. These findings are highly beneficial for better landscape design and management, improving the public’s perception and satisfaction with landscapes. They draw relevance from our investigation into the restorative effects of richly colored plants in autumn compared to bare tree plants.

#### 4.1.2 Autumn bare tree landscapes also contribute to recovery

Previous research has shown that bare tree landscapes significantly influence psychological relaxation and emotional improvement in individuals [45], aligning with our study’s results. Even in seasons where plant colors are relatively monochromatic, bare tree landscapes still exhibit notable effects on mental recovery. Moreover, both barren tree and withered branch landscapes can offer benefits, especially when adding dynamic bird songs and small animal activities to the barren tree landscape. This can have a positive impact on individuals’ physical and mental recovery during tranquil moments in late autumn and has gained recognition from most respondents.

Studies suggest that natural environments like urban areas, forests, and campuses that support human mental recovery require diverse landscape types, and barren tree landscapes can serve as an integral part of seasonal dynamic changes, enhancing their effectiveness [44]. Barren tree landscapes in nature can be seen as "natural barriers," aiding people in better focusing on their sensations and emotional experiences, thereby alleviating anxiety and stress [46]. Within barren tree landscapes, due to their monochromatic appearance and seasonal variations, they hold certain value.

Research has also found that barren tree landscapes, to some extent, can alleviate human psychological stress and fatigue, attributable to their landscape characteristics [47]. This implies that during autumn, especially post-fall foliage, landscapes characterized by monochromatism or barren features can still appeal to specific audiences. Monotonous landscapes, bare trees, and post-fall scenery continue to play a role in aesthetics and recovery, as confirmed in psychological and physiological research [13].

### 4.2 Differential effects of perceptual dimensions in virtual environments on human recovery

In terms of psychological recovery, the limitations of virtual environments lead to differences in the effects of viewing forests in various dimensions compared to real-life situations, consistent with previous research [6]. Our study not only compared the richly colored autumn landscapes with monochromatic ones but also delved into the impact of different perceptual dimension stimuli on participants’ health effects.

Some studies in virtual scenarios combine visual and auditory elements, evaluating people’s psychological states by measuring physiological indicators, and find that regardless of the landscape characteristics, visually and aurally combined virtual scenarios appear to have a positive impact on individuals, increasing feelings of physical and emotional relaxation and pleasure. This suggests that visually and aurally combined virtual scenarios may have positive effects, and multisensory therapies may be more significant relative to single sensations. However, in this experiment, psychological questionnaire results tended to show more pronounced changes with single auditory stimulation, which may be related to our material selection and quantity [20, 48].

Previous research has indicated that multisensory dimensions can present better health effects [23]. Although our study results slightly differ, this reminds us that multi-sensory virtual experiences may offer a broader and more profound sensory experience, thus having a more significant impact on health effects. This discovery has sparked interest in further research and application of multisensory stimuli in virtual environment design.

### 4.3 Uniqueness and contributions of the study

Differing from prior studies, our research focuses on exploring the differences in the restorative effects of richly colored autumn landscapes and bare tree landscapes in virtual environments, particularly after the introduction of multisensory stimuli. Our study emphasizes the following aspects:

Landscape Research in Virtual Environments: In contrast to previous research, we shift our attention to the restorative effects of landscapes in virtual environments, offering a fresh perspective to the research field.

Comparison between Richly Colored Autumn Landscapes and Monochromatic Landscapes: Our research fills a knowledge gap that previous studies overlooked concerning the restorative effects of bare tree landscapes during the autumn season. This enables us to gain a more comprehensive understanding of the impact of landscapes in different seasons.

Multisensory Dimensions: We investigate various sensory modes, including single visual, single auditory, and audiovisual combinations, thereby expanding the scope of previous research and delving deeper into the role of multisensory stimuli in landscape restorative effects.

In conclusion, our research broadens the domain of landscape restorative effects, examining differences across various dimensions, especially in the context of bare tree landscapes, providing valuable insights for future research and landscape design.

## 5 Limitations

This study explores the restorative effects of various autumn landscapes in a virtual environment across multiple dimensions, combining the analysis of physiological and psychological indicators. However, it is important to note that this study has some limitations:

Limited Scope: This experiment exclusively investigates the restorative effects of different autumn landscapes in a virtual environment, while the effects of real-world landscapes may be influenced by seasonal and regional variations. Future research could broaden the scope, considering landscape effects under various conditions.

Further Exploration of Other Sensory Dimensions: While this study primarily focuses on visual and auditory perception, future investigations could delve into other sensory aspects such as olfaction and tactile sensation. Expanding research in these areas would offer a more comprehensive understanding of how landscapes impact individuals.

Despite stringent control over visual and auditory experimental conditions in this study, subtle differences might exist between the visual and auditory groups in terms of material collection for experiments, laboratory setups, and testing methods. Hence, future research might consider segregating the visual and auditory groups into separate laboratory environments or conducting experiments at different time intervals to minimize potential mutual influences between the two groups.

Regional and Seasonal Variations: The materials for this experiment were gathered on a campus setting, and variations in landscapes and plant species may differ significantly in various regions and seasons. Future research should take regional and seasonal factors into comprehensive consideration by studying a wider range of landscapes and plant species.

Experiment Duration Limitation: The stimulation duration in the virtual environment was only three minutes, which may affect the accuracy of the results concerning restorative effects. Future research could contemplate extending the duration of virtual environment stimulation for a more in-depth understanding of these effects.

Comparison between Real and Virtual Environments: This study did not extensively explore the differential sensory effects in real and virtual environments. Future research can delve deeper into comparing these two, aiming for a more profound understanding of their similarities and differences. Additionally, prior to the experiment, this study involved stress tests, such as typing and other laborious tasks, to enhance performance. However, we did not conduct control tests without stress to evaluate performance differences. This is a direction for further in-depth investigation. These improvements will contribute to a more comprehensive comprehension of disparities between real and virtual environments and enhance the scientific rigor and credibility of the study.

## 6 Conclusion and design recommendations

We provide the following design recommendations for landscapes and virtual environments:

Diverse Landscapes: Landscape architects and urban planners should consider the diversity of seasonal landscapes in public spaces. Providing opportunities for people to interact with a rich variety of bare tree environments can enhance well-being, particularly in situations where there is a low visitation rate for bare tree landscapes.

Multisensory Design: Incorporating multisensory elements such as soundscapes, fragrances, and tactile flora and fauna into landscape creation can optimize the therapeutic effects of the environment. This design approach aligns with our findings regarding the importance of sensory combinations.

Long-Term Experiments: Future research should encompass long-term experiments to assess the sustained impact of various landscapes on recovery. This will contribute to providing more comprehensive recommendations for landscape design.

Cultural and Contextual Factors: Landscape preferences and their effects may vary due to cultural and geographical differences. Designers should consider local cultural and environmental factors when designing landscapes.

Integration with Real Environments: While virtual environments are valuable for controlled studies, applying our findings to real-world settings is crucial for practical urban planning.

By implementing these recommendations and addressing the limitations of our research, we can further enhance the design of landscapes and virtual environments to promote human well-being and recovery. This study offers valuable insights at the intersection of nature, sensory experiences, and health, paving the way for more inclusive and restorative urban environments.

## Supporting information

S1 Table Baseline detection of participants’ brain waves. (PDF)

S2 Table Baseline psychological assessment of participants. (PDF)

S3 Table The EEG and heart rate changes of participants experiencing autumn color plants in virtual reality. (PDF)

S4 Table Psychological index changes of autumnal colored plants. (PDF)

S5 Table The changes of EEG and heart rate in response to autumnal bare tree group stimuli. (PDF)

S6 Table Psychological indicators of changes in autumn bare tree plants. (PDF)

S7 Table Physiological impact of perceived dimensions of autumn landscapes in a virtual environment on human recovery. (PDF)

S8 Table Psychological impact of perceived dimensions of autumn landscapes in a virtual environment on human recovery. (PDF)

S1 File Raw data of psychological indicators-PANAS: This file contains the raw data from pre- and post-assessments using the Positive and Negative Affect Schedule (PANAS). (XLSX)

S2 File Raw data of psychological indicators-POMS: This file contains the raw data from before and after assessments using the Profile of Mood States (POMS). (XLSX)

S3 File Raw data of psychological indicators-ROS: This file contains the pre- and post-assessment raw data from the Reality Orientation Screening (ROS). (XLSX)

S4 File Raw data of psychological indicators-SVS: This file includes raw data from pre- and post-assessment with the Schwartz Value Survey (SVS). (XLSX)

S5 File Raw physiological data: This file contains the raw pre- and post-assessment data for Electroencephalogram (EEG) and heart rate measurements. (XLSX)

S1 Data (DOCX)
