# Supplementary material for: Exploring the impact of autumn color and bare tree landscapes in virtual environments on human well-being and therapeutic effects across different sensory modalities
Source: PLoS One. 2024 Apr 18;19(4):e0301422. doi: 10.1371/journal.pone.0301422 (PMC11025894; doi:10.1371/journal.pone.0301422)
Supplement: S1 Table — (PDF) [file pone.0301422.s001.pdf]

S1 Table . Baseline detection of participants' brain waves.

		EEG (Electroencephalography)						HR		
		α 1			α 2					
		**Average value**	**Standard deviation**	***p***	**Average value**	**Standard deviation**	***p***	**Average value**	**Standard deviation**	***p***
**Color group**	**Visual group**	31650.0906	13411.8092	0.8804	21790.7848	8351.4067	0.955	76.375	11.9396	0.9327
	**Auditory group**	24598.3154	6984.4833	0.9996	23129.2112	13414.4131	0.841	74.625	18.361	0.9941
	**Audio-visual group**	30143.5621	13373.6432	0.9709	22716.6049	8718.4855	0.8843	72.125	8.593	1
**Bare Tree group**	**Visual group**	27178.689	27178.689	0.9998	22376.2711	12935.2811	0.9148	79.25	6.5629	0.6295
	**Auditory group**	27320.5032	5621.7146	0.9997	21576.8828	4389.1660	0.9659	77.375	9.3494	0.8504
	**Audio-visual group**	46047.2903	14515.8761	0.0091**	35699.7704	14299.1133	0.0066**	75.375	11.0316	0.9792

* $p < 0.05$ Significant difference

**$p < 0.01$ Extremely significant difference
