# Supplementary material for: Exploring the impact of autumn color and bare tree landscapes in virtual environments on human well-being and therapeutic effects across different sensory modalities
Source: PLoS One. 2024 Apr 18;19(4):e0301422. doi: 10.1371/journal.pone.0301422 (PMC11025894; doi:10.1371/journal.pone.0301422)
Supplement: S2 Table — (PDF) [file pone.0301422.s002.pdf]

S2 Table . Baseline psychological assessment of participants.

		POMS			PANAS			SVS			ROS		
		**Average value**	**Standard deviation**	**p**	**Average value**	**Standard deviation**	**p**	**Average value**	**Standard deviation**	**p**	**Average value**	**Standard deviation**	**p**
**Color group**	**Visual group**	81.25	18.04	0.9979	46.25	9.85	0.3946	36.875	7	0.4778	21.5	3	0.9998
	**Auditory group**	85.5	16.05	0.8449	42	8.63	0.9048	30.5	7.28	0.9999	21.5	3.7	0.9997
	**Audio-visual group**	81.125	13.79	0.9999	38.75	14.7	0.9998	33.5	7.09	0.9635	20.875	8.51	0.9996
**Bare Tree group**	**Visual group**	79.875	8.61	>0.9999	37.25	6.65	0.9999	34	9.48	0.8343	21.5	2.5	0.998
	**Auditory group**	88.125	14.54	0.7396	43.625	9.78	0.7418	33.375	10.05	0.9925	22	3.6	0.9789
	**Audio-visual group**	78.375	18.11	0.9983	34.5	8.74	0.8781	30.375	8.99	0.9997	20.5	2.14	0.9876
