# Supplementary material for: Exploring the impact of autumn color and bare tree landscapes in virtual environments on human well-being and therapeutic effects across different sensory modalities
Source: PLoS One. 2024 Apr 18;19(4):e0301422. doi: 10.1371/journal.pone.0301422 (PMC11025894; doi:10.1371/journal.pone.0301422)
Supplement: S3 Table — (PDF) [file pone.0301422.s003.pdf]

S3 Table . The EEG and heart rate changes of participants experiencing autumn color plants in virtual reality.

			EEG (electroencephalogram) machine				HR	
			α 1		α 2			
			Pre-test	Post-test	Pre-test	Post-test	Pre-test	Post-test
Blank control group		Average value	25622.6782	22274.7877	20024.4577	18635.4551	74.88	74.25
		Standard deviation	7216.6759	6602.9302	4195.6022	5696.4333	5.249	3.845
		t	1.028		0.588		0.424	
		p	0.338		0.575		0.685	
		Effect size	0.13751		0.23523		0.06831	
Color group	Visual group	Average value	34288.7881	32305.5531	22883.3373	22094.7406	74.00	71.75
		Standard deviation	13719.0806	11874.4549	7722.9388	6435.0590	12.095	12.384
		t	0.739		0.373		1.131	
		p	0.484		0.720		0.295	
		Effect size	0.07706		0.05539		0.09152	
	Auditory group	Average value	25933.2206	20755.2848	19019.2335	27483.9139	59.25	56.25
		Standard deviation	4996.7091	12048.5939	4268.4253	28483.7151	40.103	38.202
		t	1.506		-0.873		2.366	
		p	0.176		0.412		0.050	
		Effect size	0.27026		0.20347		0.03827	
	Audio-visual group	Average value	33667.0740	35447.0077	26134.0499	28095.4862	70.00	67.75
		Standard deviation	14415.2728	24267.2688	11891.0123	16549.6647	11.187	9.067
		t	-0.307		-0.563		0.874	
		p	0.768		0.591		0.411	
		Effect size	0.08918		0.0679		0.10982	

* $p < 0.05$ Significant difference

** $p < 0.01$ Extremely significant difference
