# Supplementary material for: Exploring the impact of autumn color and bare tree landscapes in virtual environments on human well-being and therapeutic effects across different sensory modalities
Source: PLoS One. 2024 Apr 18;19(4):e0301422. doi: 10.1371/journal.pone.0301422 (PMC11025894; doi:10.1371/journal.pone.0301422)
Supplement: S4 Table — (PDF) [file pone.0301422.s004.pdf]

S4 Table . Psychological index changes of autumnal colored plants.

			SVS		ROS		PANAS		POMS	
			Pre-test	Post-test	Pre-test	Post-test	Pre-test	Post-test	Pre-test	Post-test
Blank control group		Average value	29.88	31.13	18.75	16.63	17.00	10.63	-21.12	-29.12
		Standard deviation	6.707	5.915	5.471	4.406	9.739	6.413	25.754	24.544
		t	0.886		1.8338		1.208		1.195	
		p	0.405		0.109		0.226		0.271	
		Effect size	0.09836		0.2087		0.15672		0.15703	
Color group	Visual group	Average value	31.13	37.25	15.38	18 .25	14.63	19.88	-10.87	1.25
		Standard deviation	7.039	8.190	4.104	3.327	6.989	7.791	22.203	13.520
		t	-1.634		-2.556		-3.111		-2.640	
		p	0.146		0.038*		0.017*		0.033*	
		Effect size	0.2646		0.355858		0.33428		0.3131	
	Auditory group	Average value	28.50	33.63	13.00	16.50	12.38	19.25	-19.25	-6.50
		Standard deviation	5.127	6.589	4.629	4.276	10.183	9.498	20.169	17.639
		t	-2.900		-3.704		-2.508		-2.734	
		p	0.023*		0.008**		0.041*		0.029*	
		Effect size	0.3985		0.36555		0.32939		0.31891	
	Audio-visual group	Average value	31.50	35.75	16.00	18.50	8.50	17.00	-24.75	-8.25
		Standard deviation	9.621	7.106	7.559	6.279	9.986	9.739	20.968	16.985
		t	-2.335		-1.498		-3.149		-2.656	
		p	0.052		0.178		0.016*		0.033*	
		Effect size	0.24368		0.17705		0.39572		0.39687	

* $p<0.05$ Significant difference

**$p<0.01$ Extremely significant difference
