# Supplementary material for: Exploring the impact of autumn color and bare tree landscapes in virtual environments on human well-being and therapeutic effects across different sensory modalities
Source: PLoS One. 2024 Apr 18;19(4):e0301422. doi: 10.1371/journal.pone.0301422 (PMC11025894; doi:10.1371/journal.pone.0301422)
Supplement: S5 Table — (PDF) [file pone.0301422.s005.pdf]

S5 Table .The changes of EEG and Heart rate in response to autumnal bare tree group stimuli.

			EEG				HR	
			α 1		α 2			
			Pre-test	Post-test	Pre-test	Post-test	Pre-test	Post-test
Blank control group		Average value	25622.6782	22274.7877	20024.4577	18635.4551	74.88	74.25
		Standard deviation	7216.6759	6602.9302	4195.6022	5696.4333	5.249	3.845
		t	1.028		0.588		0.424	
		p	0.338		0.575		0.685	
		Effect size	0.23523		0.13751		0.06831	
Bare Tree group	Visual group	Average value	24382.4491	55882.7853	18062.4458	38173.5729	80.63	82.50
		Standard deviation	7710.2198	41181.2898	4409.7635	28066.7236	5.655	7.483
		t	-2.464		-2.164		-1.135	
		p	0.043*		0.067		0.294	
		Effect size	0.46942		0.4476		0.1396	
	Auditory group	Average value	27247.4289	26228.0041	23172.8329	37735.4491	77.13	77.13
		Standard deviation	4516.5561	11571.3186	6879.0406	21074.1357	8.254	8.114
		t	0.279		-2.229		0.000	
		p	0.788		0.061		1.000	
		Effect size	0.05793		0.42127		0	
	Audio-visual group	Average value	46067.7001	42779.7213	37682.8713	33416.8398	76.25	76.25
		Standard deviation	17862.6821	26524.8292	18481.9835	26034.4261	9.036	7.402
		t	0.384		0.393		0.000	
		p	0.712		0.706		1.000	
		Effect size	0.07251		0.09406		0	

* $p < 0.05$ Significant difference

** $p < 0.01$ Extremely significant difference
