# Supplementary material for: Exploring the impact of autumn color and bare tree landscapes in virtual environments on human well-being and therapeutic effects across different sensory modalities
Source: PLoS One. 2024 Apr 18;19(4):e0301422. doi: 10.1371/journal.pone.0301422 (PMC11025894; doi:10.1371/journal.pone.0301422)
Supplement: S6 Table — (PDF) [file pone.0301422.s006.pdf]

S6 Table .Psychological indicators of changes in autumn bare tree plants.

			SVS		ROS		PANAS		POMS	
			Pre-test	Post-test	Pre-test	Post-test	Pre-test	Post-tes t	Pre-tes t	Post-test
Blank control group		Average value	31.13	29.88	18.75	16.63	17.00	10.63	-21.12	-29.12
		Standard deviation	6.707	5.915	5.471	4.406	9.739	6.413	25.754	24.544
		t	0.886		1.8338		1.208		1.195	
		p	0.405		0.109		0.226		0.271	
		Effect size	0.09836		0.2087		0.15672		0.15703	
Bare Tree group	Visual group	Average value	33.88	36.38	15.38	19.00	16.88	20.63	-15.50	-6.37
		Standard deviation	9.478	10.954	4.307	6.071	7.376	8.297	20.149	16.248
		t	-1.379		-2.662		-1.426		-3.160	
		p	0.210		0.032*		0.197		0.016*	
		Effect size	0.12114		0.32519		0.23232		0.242	
	Auditory group	Average value	34.63	37.50	15.25	18.63	12.38	19.25	-28.25	-14.12
		Standard deviation	6.545	9.562	5.874	5.423	9.546	10.964	28.050	27.792
		t	-1.600		-2.216		-1.865		-3.889	
		p	0.154		0.062		0.104		0.006**	
		Effect size	0.17251		0.28643		0.31693		0.2453	
	Audio-visual group	Average value	30.38	34.38	15.00	16.63	9.75	9.88	-17.75	-20.25
		Standard deviation	9.226	8.700	5.555	5.854	9.331	11.569	9.692	14.772
		t	-2.605		-0.684		-0.044		0.755	
		p	0.035*		0.516		0.966		0.475	
		Effect size	0.21769		0.14139		0.00618		0.09956	

* $p < 0.05$ Significant difference

** $p < 0.01$ Extremely significant difference
