# Supplementary material for: Exploring the impact of autumn color and bare tree landscapes in virtual environments on human well-being and therapeutic effects across different sensory modalities
Source: PLoS One. 2024 Apr 18;19(4):e0301422. doi: 10.1371/journal.pone.0301422 (PMC11025894; doi:10.1371/journal.pone.0301422)
Supplement: S7 Table — (PDF) [file pone.0301422.s007.pdf]

S7 Table . Physiological impact of perceived dimensions of autumn landscapes in a virtual environment on human recovery.

			α 1		α 2		HR	
			Pre-test	Post-test	Pre-test	Post-test	Pre-test	Post-test
Blank control group		Average value	25622.6782	22274.7877	20024.4577	18635.4551	74.88	74.25
		Standard deviation	7216.6759	6602.9302	4195.6022	5696.4333	5.249	3.845
		t	1.028		0.588		0.424	
		p	0.338		0.575		0.685	
		Effect size	0.23523		0.13751		0.06831	
Visual group	Color group	Average value	34288.7881	32305.5531	22883.3373	22094.7406	74.00	71.75
		Standard deviation	13719.0806	11874.4549	7722.9388	6435.0590	12.095	12.384
		t	0.739		0.373		1.131	
		p	0.484		0.720		0.295	
		Effect size	0.07706		0.05539		0.09152	
	Bare Tree group	Average value	24382.4491	55882.7853	18062.4458	38173.5729	80.63	82.50
		Standard deviation	7710.2198	41181.2898	4409.7635	28066.7236	5.655	7.483
		t	-2.464		-2.164		-1.135	
		p	0.043*		0.067		0.294	
		Effect size	0.46942		0.4476		0.1396	
Auditory group	Color group	Average value	25933.2206	20755.2848	19019.2335	27483.9139	59.25	56.25
		Standard deviation	4996.7091	12048.5939	4268.4253	28483.7151	40.103	38.202
		t	1.506		-0.873		2.366	
		p	0.176		0.412		0.050	
		Effect size	0.27026		0.20347		0.03827	
	Bare Tree group	Average value	27247.4289	26228.0041	23172.8329	37735.4491	77.13	77.13
		Standard deviation	4516.5561	11571.3186	6879.0406	21074.1357	8.254	8.114
		t	0.279		-2.229		0.000	
		p	0.788		0.061		1.000	

		**Effect size**	0.05793		0.42127		0	
**Audio-visual group**	**Color group**	**Average value**	33667.0740	35447.0077	26134.0499	28095.4862	70.00	67.75
		**Standard deviation**	14415.2728	24267.2688	11891.0123	16549.6647	11.187	9.067
		t	-0.307		-0.563		0.874	
		p	0.768		0.591		0.411	
		**Effect size**	0.08918		0.0679		0.10982	
	**Bare Tree group**	**Average value**	46067.7001	42779.7213	37682.8713	33416.8398	76.25	76.25
		**Standard deviation**	17862.6821	26524.8292	18481.9835	26034.4261	9.036	7.402
		t	0.384		0.393		0.000	
		p	0.712		0.706		1.000	
		**Effect size**	0.07251		0.09406		0	

* $p < 0.05$ Significant difference

** $p < 0.01$ Extremely significant difference
