# Supplementary material for: Exploring the impact of autumn color and bare tree landscapes in virtual environments on human well-being and therapeutic effects across different sensory modalities
Source: PLoS One. 2024 Apr 18;19(4):e0301422. doi: 10.1371/journal.pone.0301422 (PMC11025894; doi:10.1371/journal.pone.0301422)
Supplement: S1 Data — (DOCX) [file pone.0301422.s014.docx]

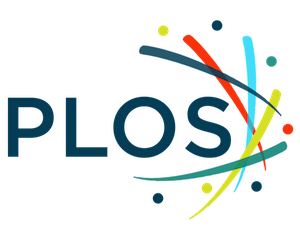


Study Protocol Article Template

**Title**  Exploring the impact of autumn color and bare tree landscapes in virtual environments on human well-being and therapeutic effects across different sensory modalities

**Metadata**

Funding:

This research was funded by the Key Research and Development Plan of Shaanxi Province, Grant No. 2020NY-105; Art Project of National Social Science Foundation of China, Grant No. 21BH156

Competing interests Data:

The authors declare that the research was conducted in the absence of any commercial or financial relationships that could be construed as a potential conflict of interest.

Availability:

All data, including the raw datasets, provided in the supporting information, are accessible

# **Abstract:**In recent years, there has been a growing awareness of the potential health benefits of the natural environment for human well-being. Given the fast-paced nature of contemporary lifestyles, research into the use of virtual environments as a means to provide various seasonal landscapes has gained increasing importance.Objective: The aim of this study is to investigate the impact of different sensory modes on the preferences and therapeutic effects of virtual autumn landscapes on university campuses.Methods: In this study, 320 participants, with an average age of 21.11 years (±1.21 years), were exposed to virtual environments featuring autumn color landscapes and bare tree landscapes using visual, auditory, and combined conditions. A control group was included for comparison. Differences in participants' physiological indicators (EEG, heart rate) and psychological measures (POMS, PANAS, SVS, ROS) were analyzed, with the use of the Holm correction (P < 0.05).Results:(1) Autumn virtual landscapes with color had a superior therapeutic effect. (2) There were significant differences in the therapeutic effects of different sensory modes within the same season's landscape categories, suggesting that incorporating additional sensory dimensions may enhance therapeutic outcomes.Conclusion:Based on the study's findings, we recommend that when designing therapeutic environments, attention should be given to seasonal variations and the integration of various sensory modes to optimize therapeutic results.

# **Introduction**

The study focuses on three key areas: the impact of campus landscapes on individual recovery, the influence of autumn landscape elements on well-being[1-3], and the role of sensory dimensions in promoting physical and mental recovery. Additionally, it delves into the application of Virtual Reality (VR) technology in landscape restoration. These areas are discussed within the context of established theories like Attention Restoration Theory (ART) and Stress Reduction Theory (SRT) and draw upon the works of prominent scholars in the field.

The Impact of Campus Landscapes

Natural environments within campus landscapes play a significant role in nurturing physical and mental well-being. They facilitate emotional experiences, stress reduction, and heightened satisfaction among university students. While extensive research exists on the relationship between campus landscapes and well-being, further exploration is needed to understand how seasonal variations in natural campus environments affect healing outcomes[4-15].

The Influence of Autumn Landscape Elements

Traditional emphasis on the restorative benefits of green vegetation often overlooks the impact of seasonal variations on individual preferences. During autumn, vibrant colors and barren tree landscapes gain prominence, influencing restoration. This study aims to elucidate the differential effects between autumn-colored plants and bare trees, emphasizing the importance of post-autumn landscapes in the restoration process.

The Influence of Sensory Dimensions

The study departs from traditional visual-centric research and acknowledges the multidimensional nature of sensory perception. It highlights the significance of exploring visual, auditory, and olfactory experiences in understanding the therapeutic effects of landscape vegetation. Shifting from single sensory experiences to multifaceted sensory dimensions helps to comprehend variations in physical and mental recovery among participants[16-18].

The Application of VR Technology

Given the limitations of field surveys, the study recognizes the importance of VR technology in simulating natural environments. The integration of VR-based natural landscape therapy aids in understanding people's preferences and stress reduction potential. The research particularly emphasizes the restorative outcomes of virtual landscape experiences and their relevance in stress reduction[19-25].

Research Objectives and Hypotheses

Rooted in established theories, the study aims to explore the therapeutic effects of autumn landscapes and bare tree environments in virtual environments. It postulates hypotheses suggesting the restorative impact of bare tree landscapes and the influence of sensory stimuli on physiological and psychological responses.

This structured introduction provides a comprehensive overview of the study's objectives, hypotheses, and the anticipated contributions to the existing body of literature on landscape restoration and human well-being[26-28].

# Materials and Methods

# Aim, Design, and Setting of the Study:

The study aimed to explore the impact of visual and auditory stimuli, specifically focusing on autumn landscapes and their effects on participants' mental and physical states.

The experimental design included random recruitment of 320 volunteers from Northwest A&F University, with different experimental groups: control, visual, auditory, and audiovisual[29].

# Sample Size, Inclusion, and Exclusion Criteria:

320 volunteers were recruited in December 2022 from various disciplines at the university.

Criteria included absence of mental disorders, normal vision and hearing, and an age average of 21.11 years[30-34].

# Characteristics of Participants and Selection Process:

Recruitment was random and stringent ethical standards were followed. Volunteers were briefed on pre-experiment restrictions and assigned unique identifiers.

# Description of Processes, Interventions, and Comparisons:

Different experimental groups were used to assess the effects of visual, auditory, and combined stimuli on participants' mental states.

# Outcomes, Measurement Methods:

The study measured participants' responses to different stimuli through VR videos and high-resolution auditory recordings.

Evaluation criteria were established by landscape architecture experts for both visual and auditory stimuli[35-37].

# Data Management Plans:

Data were collected and evaluated by experts using specific criteria for assessment.

# Safety Considerations:

Precautions were taken to avoid external interference and maintain a comfortable indoor environment for the experiment.

# Type of Data and Statistical Analyses Planned:

This study performed statistical processing of all data using Microsoft Excel 2016 and conducted statistical analysis using SPSS 22.0 [38]. Physiological and psychological responses of participants were subjected to one-way ANOVA and paired t-tests under different stimulus conditions (color group, bare tree group, single-dimensional to multi-dimensional conditions, and control group). Holm correction was applied in the statistical analysis (P<0.05) to ensure statistical significance of results. Cohen’s d was used to represent the effect size of t-tests [39]. Finally, data visualization was done using Photoshop 2019 and Prismchs[40-41].

# Ethical Considerations and Declarations:

The study strictly adhered to ethical standards set by the Psychological Center at Northwest A&F University.

# Status and Timeline of the Study:

The study was conducted in December 2022.

# Discussion

Limitations in the Study Design

Our research focused on the influence of seasonal landscape colors and perceptual dimensions in virtual environments, but there are limitations to consider:

Ignored Seasonal and Regional Variations:

We only explored virtual autumn landscapes, potentially overlooking variations across different seasons and regions. Future studies should encompass broader landscape changes[42].

Limited Duration of Virtual Stimulation:

The stimuli in the virtual environment lasted only three minutes, possibly restricting a deeper observation of recovery effects. Extending this duration might offer more profound insights[43].

Further Investigation of Sensory Dimensions Needed:

While our study emphasized visual and auditory perceptions, future research could explore additional sensory dimensions like smell and touch for a comprehensive understanding of landscape impact.

Comparison between Real and Virtual Environments:

We didn't fully explore differences in sensory dimensions between real and virtual environments. Future research could extensively compare these to deeply understand their similarities and differences[44-48].

Constraints in Stress Testing:

Stress tests, such as typing before the experiment to enhance performance, were conducted. However, we didn't have a control test without stress to evaluate performance differences. This area requires further investigation.

Dissemination Plans: The study results, encompassing diverse landscape effects on recovery, call for dissemination through scholarly publications and conference presentations. Further, dissemination among urban planners and landscape architects would facilitate the integration of our findings into practical applications for public spaces.

Handling Study Amendments and Termination:

Although this study didn’t encounter amendments or termination, managing such instances is crucial in research. Potential amendments, like methodological improvements or unforeseen circumstances, should be addressed through ethical review and appropriate documentation. Termination of a study, if necessary, should strictly adhere to ethical guidelines and involve comprehensive reporting.

# Authors’ contributions Acknowledgements

The authors would like to express their gratitude to all those who have contributed to the completion of this study.

We would also like to thank the individuals who shared their valuable time and expertise for the purpose of this study. Their contributions were instrumental in shaping the direction of our research and enhancing the quality of the data.

Furthermore, we acknowledge the participation of all study participants who enabled us to collect and analyze data relevant to this research. Without their time and effort, this study would not be possible.

In conclusion, we are immensely grateful to all those who have contributed to this research, and we extend our heartfelt thanks to each and every one of them

**Supporting Information**

S1 Table. Baseline detection of participants' brain waves.

S2 Table. Baseline psychological assessment of participants.

S3 Table. The EEG and heart rate changes of participants experiencing autumn color plants in virtual reality.

S4 Table. Psychological index changes of autumnal colored plants.

S5 Table. The changes of EEG and Heart rate in response to autumnal bare tree group stimuli.

S6 Table. Psychological indicators of changes in autumn bare tree plants.

S7 Table. Physiological indicators changes from single to multi-dimensional perspective

S8 Table. Psychological indicator changes from a single perspective to a multi-dimensional perspective.

**References**

1. Kaplan S. The restorative benefits of nature: Toward an integrative framework. Journal of environmental psychology. 1995;15(3):169-82. doi: 10.1002/jclp.10229.

2. Liu Q, Zhang Y, Lin Y, You D, Zhang W, Huang Q, et al. The relationship between self-rated naturalness of university green space and students’ restoration and health. Urban Forestry & Urban Greening. 2018;34:259-68. doi: 10.1016/j.ufug.2018.07.008.

3. Lina G. The Impact of Campus Street Tree Landscape on Stress Relief of College Students [M.A. thesis]: Northwest A&F University; 2020.

4. Lee J, Tsunetsugu Y, Takayama N, Park B-J, Li Q, Song C, et al. Influence of forest therapy on cardiovascular relaxation in young adults. Evidence-based complementary and alternative medicine. 2014;2014. doi: 10.1155/2014/834360.

5. Ulrich RS. View through a window may influence recovery from surgery. science. 1984;224(4647):420-1. doi: 10.1126/science.6143402.

6. Berman MG, Jonides J, Kaplan S. The cognitive benefits of interacting with nature. Psychological science. 2008;19(12):1207-12. doi: 10.1111/j.1467-9280.2008.02225.x.

7. Hakamata Y, Lissek S, Bar-Haim Y, Britton JC, Fox NA, Leibenluft E, et al. Attention bias modification treatment: a meta-analysis toward the establishment of novel treatment for anxiety. Biological psychiatry. 2010;68(11):982-90. doi: 10.1016/j.biopsych.2010.07.021.

8. Junge X, Schüpbach B, Walter T, Schmid B, Lindemann-Matthies P. Aesthetic quality of agricultural landscape elements in different seasonal stages in Switzerland. Landscape and Urban Planning. 2015;133:67-77. doi: 10.1016/j.landurbplan.2014.09.010.

9. Wang R, Zhao J. Effects of evergreen trees on landscape preference and perceived restorativeness across seasons. Landscape Research. 2020;45(5):649-61. doi: 10.1080/01426397.2019.1699507.

10. Kuper R. Effects of flowering, foliation, and autumn colors on preference and restorative potential for designed digital landscape models. Environment and Behavior. 2020;52(5):544-76. doi: 10.1177/0013916518811424.

11. Bielinis E, Takayama N, Boiko S, Omelan A, Bielinis L. The effect of winter forest bathing on psychological relaxation of young Polish adults. Urban Forestry & Urban Greening. 2018;29:276-83. doi: 10.1016/j.ufug.2017.12.006.

12. Bielinis E, Janeczko E, Takayama N, Zawadzka A, Słupska A, Piętka S, et al. The effects of viewing a winter forest landscape with the ground and trees covered in snow on the psychological relaxation of young Finnish adults: A pilot study. PLoS One. 2021;16(1):e0244799. doi: 10.1371/journal.pone.0244799.

13. Berto R. The role of nature in coping with psycho-physiological stress: A literature review on restorativeness. Behavioral sciences. 2014;4(4):394-409. doi: 10.3390/bs4040394.

14. Guo L-N, Zhao R-L, Ren A-H, Niu L-X, Zhang Y-L. Stress recovery of campus street trees as visual stimuli on graduate students in autumn. International Journal of Environmental Research and Public Health. 2020;17(1):148. doi: 10.3390/ijerph17010148.

15. Guan C, Song J, Keith M, Zhang B, Akiyama Y, Da L, et al. Seasonal variations of park visitor volume and park service area in Tokyo: A mixed-method approach combining big data and field observations. Urban Forestry & Urban Greening. 2021;58:126973. doi: 10.1016/j.ufug.2020.126973.

16. Bradley MM, Lang PJ. Affective reactions to acoustic stimuli. Psychophysiology. 2000;37(2):204-15. doi: 10.1017/S0048577200990012.

17. Annerstedt M, Jönsson P, Wallergård M, Johansson G, Karlson B, Grahn P, et al. Inducing physiological stress recovery with sounds of nature in a virtual reality forest—Results from a pilot study. Physiology & behavior. 2013;118:240-50. doi: 10.1016/j.physbeh.2013.05.023.

18. Kardan O, Demiralp E, Hout MC, Hunter MR, Karimi H, Hanayik T, et al. Is the preference of natural versus man-made scenes driven by bottom–up processing of the visual features of nature? Frontiers in psychology. 2015;6:471. doi: 10.3389/fpsyg.2015.00471.

19. Li K, Zhai Y, Dou L, Liu J. A preliminary exploration of landscape preferences based on naturalness and visual openness for college students with different moods. Frontiers in Psychology. 2021;12:629650. doi: 10.3389/fpsyg.2021.629650.

20. Ma J, Zhao D, Xu N, Yang J. The effectiveness of immersive virtual reality (VR) based mindfulness training on improvement mental-health in adults: A narrative systematic review. Explore. 2023;19(3):310-8.

21. Xu J, Jo H, Noorbhai L, Patel A, Li A. Virtual mindfulness interventions to promote well-being in adults: A mixed-methods systematic review. Journal of affective disorders. 2022;300:571-85.

22. Bradburn NM, Rips LJ, Shevell SK. Answering autobiographical questions: The impact of memory and inference on surveys. Science. 1987;236(4798):157-61. doi: 10.1126/science.3563494.

23. Zajonc RB. Feeling and thinking: Preferences need no inferences. American psychologist. 1980;35(2):151-75. doi: 10.1037/0003-066X.35.2.151.

24. Jensen O, Tesche CD. Frontal theta activity in humans increases with memory load in a working memory task. European journal of Neuroscience. 2002;15(8):1395-9. doi: 10.1046/j.1460-9568.2002.01975.x.

25. Yeh S-C, Li Y-Y, Zhou C, Chiu P-H, Chen J-W. Effects of virtual reality and augmented reality on induced anxiety. IEEE Transactions on Neural Systems and Rehabilitation Engineering. 2018;26(7):1345-52. doi: 10.1109/TNSRE.2018.2844083.

26. Kaplan R, Kaplan S. The experience of nature: A psychological perspective: Cambridge university press; 1989.

27. Ulrich RS. Aesthetic and affective response to natural environment. Behavior and the natural environment. Springer; 1983. p. 85-125.

28. Mengqiu W. Campus plant color research on the impact of physical and mental health among college students [M.A. thesis]: Northwest A&F University; 2018.

29. Tourangeau R, Yan T. Sensitive questions in surveys. Psychological bulletin. 2007;133(5):859. doi: 10.1037/0033-2909.133.5.859.

30. Tugade MM, Fredrickson BL, Feldman Barrett L. Psychological resilience and positive emotional granularity: Examining the benefits of positive emotions on coping and health. Journal of personality. 2004;72(6):1161-90. doi: 10.1111/j.1467-6494.2004.00294.x.

31. Wang X, Shi Y, Zhang B, Chiang Y. The influence of forest resting environments on stress using virtual reality. International journal of environmental research and public health. 2019;16(18):3263. doi: 10.3390/ijerph16183263.

32. Hankins TC, Wilson GF. A comparison of heart rate, eye activity, EEG and subjective measures of pilot mental workload during flight. Aviation, space, and environmental medicine. 1998;69(4):360-7.

33. Kropotov JD. Quantitative EEG, event-related potentials and neurotherapy: Academic Press; 2010.

34. Gao T, Zhang T, Zhu L, Gao Y, Qiu L. Exploring psychophysiological restoration and individual preference in the different environments based on virtual reality. International journal of environmental research and public health. 2019;16(17):3102. doi: 10.3390/ijerph16173102.

35. Kamarck TW, Shiffman SM, Smithline L, Goodie JL, Paty JA, Gnys M, et al. Effects of task strain, social conflict, and emotional activation on ambulatory cardiovascular activity: daily life consequences of recurring stress in a multiethnic adult sample. Health Psychology. 1998;17(1):17-29. doi: 10.1037/0278-6133.17.1.17.

36. Spielberger CD. Review of Profile of Mood States. 1972;3(4):387-8. doi: 10.1037/h0020742.

37. Crawford JR, Henry JD. The Positive and Negative Affect Schedule (PANAS): Construct validity, measurement properties and normative data in a large non‐clinical sample. British journal of clinical psychology. 2004;43(3):245-65. doi: 10.1348/0144665031752934.

38. Korpela KM, Ylén M, Tyrväinen L, Silvennoinen H. Determinants of restorative experiences in everyday favorite places. Health & place. 2008;14(4):636-52. doi: 10.1016/j.healthplace.2007.10.008.

39. Ryan RM, Frederick C. On energy, personality, and health: Subjective vitality as a dynamic reflection of well‐being. Journal of personality. 1997;65(3):529-65. doi: 10.1111/j.1467-6494.1997.tb00326.x.

40. Field A. Discovering statistics using IBM SPSS statistics: sage; 2013.

41. Rosenthal R, Cooper H, Hedges L. Parametric measures of effect size. The handbook of research synthesis. 1994;621(2):231-44.

42. Berto R, Baroni MR, Zainaghi A, Bettella S. An exploratory study of the effect of high and low fascination environments on attentional fatigue. Journal of environmental psychology. 2010;30(4):494-500. doi: 10.1016/j.jenvp.2009.12.002.

43. Berto R. Exposure to restorative environments helps restore attentional capacity. Journal of environmental psychology. 2005;25(3):249-59. doi: 10.1016/j.jenvp.2005.07.001.

44. Lin W, Mu Y, Zhang Z, Wang J, Diao X, Lu Z, et al. Research on cognitive evaluation of forest color based on visual behavior experiments and landscape preference. Plos one. 2022;17(11):e0276677. doi: 10.1371/journal.pone.0276677.

45. Janeczko E, Bielinis E, Tiarasari U, Woźnicka M, Kędziora W, Przygodzki S, et al. How dead wood in the forest decreases relaxation? the effects of viewing of dead wood in the forest environment on psychological responses of young adults. Forests. 2021;12(7):871. doi: 10.3390/f12070871.

46. Herzog TR. A cognitive analysis of preference for urban nature. Journal of environmental Psychology. 1989;9(1):27-43. doi: 10.1016/S0272-4944(89)80024-6.

47. Dijkstra K, Pieterse ME, Pruyn A. Stress-reducing effects of indoor plants in the built healthcare environment: The mediating role of perceived attractiveness. Preventive medicine. 2008;47(3):279-83. doi: 10.1016/j.ypmed.2008.01.013.

48. Asiain J, Braun M, Roussos AJ. Virtual reality as a psychotherapeutic tool: current uses and limitations. British Journal of Guidance & Counselling. 2022;50(1):1-28. doi: 10.1080/03069885.2021.1885008. PubMed PMID: WOS:000646831100001.
